# Mitochondrial Targeted Antioxidant SKQ1 Ameliorates Acute Kidney Injury by Inhibiting Ferroptosis

**DOI:** 10.1155/2022/2223957

**Published:** 2022-09-22

**Authors:** Jiayu Song, Jingyi Sheng, Juan Lei, Weihua Gan, Yunwen Yang

**Affiliations:** ^1^Department of Pediatric Nephrology, The Second Affiliated Hospital of Nanjing Medical University, Nanjing, Jiangsu 210003, China; ^2^Nanjing Key Laboratory of Pediatrics, Children's Hospital of Nanjing Medical University, Nanjing 210008, China

## Abstract

Emerging evidence suggests that ferroptosis is highly correlated with the pathogenesis of acute kidney injury (AKI). Ferroptosis, an iron-dependent form of cell death, is manifested by a toxic accumulation of lipid peroxides and ultrastructural changes in mitochondria. We herein investigated the effect of Visomitin (SKQ1), a novel mitochondria-targeting antioxidant, on several AKI models *in vivo* and *in vitro*. Our results revealed that SKQ1 treatment greatly reversed renal outcomes in cisplatin, ischemia-reperfusion injury (IRI), or folic acid-induced AKI models. These effects were reflected in attenuated levels of renal injury biomarkers, histologic indices of tubular injury, and inflammatory infiltration in the SKQ1-treated groups. Transcriptomics analysis depicted ferroptosis signaling as the most pronounced pathway downregulated after SKQ1 treatment. Consequently, administration of SKQ1 significantly ameliorated lipid peroxide accumulation and inhibited ferroptosis in the kidneys of mice with AKI. In cultured human proximal tubule epithelial cells (HK2), SKQ1 treatment markedly mitigated cisplatin-induced mitochondrial reactive oxygen species (ROS) production, resulting in lower levels of lipid peroxidation and ferroptosis. In conclusion, SKQ1 treatment protected against ischemic- or nephrotoxic-induced AKI by inhibiting ferroptosis *in vivo* and *in vitro*. These results could facilitate a broader understanding of the interaction between mitochondrial antioxidants and ferroptotic defense mechanisms, providing a possible therapeutic strategy in AKI.

## 1. Introduction

Acute kidney injury (AKI), which is a life-threatening clinical syndrome characterized by the rapid deterioration of renal function [1], is reported to be associated with high healthcare costs, as well as increased long-term risk of morbidity and mortality [2, 3]. However, there are no effective clinically targeted treatments currently available to accelerate renal recovery, and this therefore results in subsequent chronic kidney disease (CKD). According to the etiology, AKI is divided into prerenal, renal, and postrenal caused by renal ischemia, inflammatory reactions, and acute renal tubular injury [4]. The risk factors of AKI include decreased blood flow, renal ischemic, nephrotoxic medicines, and infection [4–6]. Death of renal proximal tubular cells is the major early event in AKI, including ischemia–reperfusion injury (IRI) and nephrotoxicity, which are followed by tubular dedifferentiation, proliferation, and regeneration [7]. Numerous studies over the past decade have shown that apoptosis and modulated necrosis are the principal types of cell death in AKI [8]. Regulated necrosis is a proinflammatory death condition that results from the release of chemokines and damage-associated molecular patterns (DAMPs) from dying or dead cells [9]. Diverse types of regulated necrosis—including necroptosis, pyroptosis, and ferroptosis—have been implicated in the loss of tubular cells in AKI; and the blockade of regulated necrosis using inhibitors significantly alleviated kidney injury in animal models of AKI [10]. Thus, elucidating the regulatory mechanisms underlying tubular cell death during AKI would be useful in preventing further kidney injury and would allow for appropriate early and supportive therapy.

Ferroptosis, a novel programmed iron-dependent nonapoptotic cell death characterized by lipid peroxidation and iron accumulation [11], is highly linked to the pathogenesis of AKI [12, 13]. As an important regulator in ferroptosis, glutathione peroxidase-4 (GPX4) holds promise in protecting cells from the toxicity of ingested lipid hydroperoxides [14]; and the regulation of GPX4 expression or activity can lead to the initiation or inhibition of ferroptosis in AKI [15]. Unlike other types of regulated necrosis, excess mitochondrial fragmentation and permeabilization are closely related to ferroptosis [16]. At the morphologic level, ferroptosis is primarily manifested by condensed mitochondrial membrane densities, diminished or missing mitochondrial crista, and rupture of the outer mitochondrial membrane [17]. It is accepted that mitochondria participate in iron metabolism that involves iron utilization and catabolic and anabolic pathways [18]; and a number of studies have shown that mitochondrial defense systems not only occupy a significant role in mitochondrial lipid peroxidative detoxification but also protect against ferroptosis [19]. Nevertheless, the association between mitochondria and ferroptosis remains unclear.

Known as an antioxidant, SKQ1 plays a critical role in the treatment of pathologic conditions, including dry eye disease (DED), Alzheimer's disease, and the process of aging [20, 21]. Authors recently reported that treatment with SKQ1 allowed mice to be less prone to lipid peroxidation and show less pathologic injury than nontreated mtDNA mutated mice [22]. Another recent study revealed beneficial effects of SKQ1 in experimental diabetes by restoring the cellular antioxidant status [23]. The protection by SKQ1 of kidney IRI has been studied both *in vivo* and *in vitro*. In an experimental model of renal unilateral IRI, intraperitoneal injection (i.p.) of SKQ1 to rats markedly improved their lifespan [24]. Plotnikov et al. found that SKQ1 enhanced the survival of renal tubular epithelial cells and reduced mitochondrial fission as triggered by the ischemia/reoxygenation process *in vitro* [25]. However, the effects of SKQ1 on nephrotoxicity and the precise mechanism whereby SKQ1 acts to protect against AKI remain elusive.

Hence, the aim of the present study was to further explore the potential nephroprotective effects and mechanisms of SKQ1 against ischemic- or nephrotoxic-induced AKI. Our results suggest SKQ1 with potential in clinical treatment of AKI.

## 2. Materials and Methods

### 2.1. Mice

Adult male mice (2–3 months old and weighing 20–22 g) bred on a C57BL/6J background were obtained from the Animal Core Facility of Nanjing Medical University. All mice that were used for this study were caged at a stable temperature (21.0°C) and humidity (55.0 ± 5.0%) under a 12-hour light/dark cycle and provided regular rodent diet ad lib in specific pathogen-free facilities. Each procedure described here was conducted in compliance with the ARRIVE guidelines [26] and approved by the Animal Care and Use Committee of Nanjing Medical University.

### 2.2. Establishment of AKI Mouse Models and SKQ1 Treatment

To determine the protective effects of SKQ1 (HY-100474, MedChemExpress, Shanghai, China) on different AKI mouse models, mice were injected intraperitoneally (i.p.) with 0.2 mg/kg/d SKQ1 dissolved in vehicle (10% DMSO, 40% PEG300, 5% Tween-80, and 45% saline). For AKI induced by cisplatin, mice were assigned to four groups of six each: (1) a vehicle-treated group, in which vehicle was administered once daily both 72 h before and after a single i.p. injection of saline; (2) an SKQ1-treated group, in which SKQ1 was given once daily both 72 h before and after a single i.p. injection of saline; (3) a cisplatin-treated vehicle group, which received vehicle+CDDP (i.e., no SKQ1 pretreatment and only one injection of 25 mg/kg cisplatin); and (4) a cisplatin+SKQ1-treated group, which received SKQ1+CDDP once daily both 72 h before and after a single i.p. injection of cisplatin. For the folic acid (F7876, Sigma, St. Louis, MO)-induced AKI model, mice were randomly allocated to four groups of six: (1) a vehicle group, which received only a single i.p. injection of NaHCO_3_ (0.14 M) without SKQ1 pretreatment; (2) an SKQ1-treated group, which received SKQ1 once daily both 72 h before and after a single i.p. injection of NaHCO_3_; (3) an FA-treated vehicle group, which received vehicle+FA (i.e., no SKQ1 pretreatment and only one injection of 250 mg/kg FA); and (4) an FA+SKQ1-treated group, which received SKQ1+FA once daily both 72 h before and after a single i.p. injection of FA. For the ischemia reperfusion-induced AKI model, IRI surgery was performed as described in our previous study [27]. Mice were also allotted to four groups of six: a vehicle group (vehicle), SKQ1-treated group (SKQ1), an IRI group (vehicle+IRI), and an IRI-induced SKQ1-treated group (SKQ1+IRI). Mice in the SKQ1 treatment groups were injected i.p. with SKQ1 once daily 72 h before the IRI operation, while mice in the control group underwent the same procedure without clamping. At 72 h for the cisplatin and folic acid models or 24 h for the IRI model, we harvested serum and renal samples when the mice were prepared to be sacrificed. Clinical levels of SCr and BUN were measured with an automatic biochemical analyzer (Hitachi Ltd., USA), and specimens of kidney tissues were prepared and stored for subsequent experiments.

### 2.3. Cell Culture and Treatments

Sources and authentication of human proximal tubule epithelial cells (HK2) were from the American Type Culture Collection. Cells were cultured in DMEM/F-12 medium (319-075-CL, Thermo Fisher, MA, USA) containing 10% FBS (26170035, Gibco) under conditions of 5% CO_2_ in compressed air with high humidity at 37°C. For drug stimulation, cells were pretreated with SKQ1 for 1 h in serum- and antibiotic-free medium, and HK2 cells were then incubated with 10 *μ*g/mL cisplatin for 24 h.

### 2.4. Quantitative Real-Time PCR (qRT-PCR)

Briefly, RNA was isolated from each group of mouse tissues or cultured cells with TRIzol (9108, Takara, Osaka, Japan) and transcribed into cDNA with a reverse-transcription kit (2641A, Takara, Osaka, Japan) using the supplied protocol. QRT-PCR reactions were conducted with SYBR Green (q111-02/03, Vazyme, Nanjing, China) in accordance with a standard procedure [28] (for the primers generated by Chinese Tsingke Biotech, refer to Supplementary Table S1). The data were calculated using the 2^−*ΔΔ*Ct^ method according to previously published methods [28] where the *Δ*Ct's were normalized to the housekeeping gene *β*-actin.

### 2.5. Western Blot (WB) Analysis

Protein samples were retrieved from renal tissues or cells using the RIPA lysis buffer (P0013B, Beyotime, Shanghai, China). After preparation of the samples, we executed western immunoblotting following the manufacturer's instructions and loaded 50 *μ*g from each sample. The primary antibodies that we used were NGAL (1 : 1000, ab63929, Abcam, Cambridge, UK), KIM-1 (1 : 1000, AF1817, RD, MN, USA), cleaved caspase-3 (1 : 1000, 9664, CST, MA, USA), ATPB (1 : 1000, 17247-1-AP, Proteintech, CHI, USA), SOD2 (1 : 1000, 24127-1-AP, Proteintech, CHI, USA), myeloperoxidase (MPO) (1 : 1000, AF3667, RD, MN, US), GPX4 (1 : 1000, 14432-1-AP, Proteintech, CHI, USA), and *β*-actin (1 : 1000, 66009-1-Ig, Proteintech, CHI, USA). Secondary antibodies (A0208, A0216, Beyotime, Shanghai, China) were then added and the mixture was incubated. The immunoreactive protein signals were visualized using a Bio-Rad chemiluminescence system, with band intensity measured using ImageJ software (ImageJ 1.4, NIH, USA); and the data are shown as fold changes relative to the internal control.

### 2.6. Histologic Assessment

The kidneys from mice were dissected and fixed in 4% paraformaldehyde and embedded in paraffin. 4 *μ*m thick coronal sections were then cut from the kidneys following periodic acid-Schiff (PAS) staining according to standard protocols, and the images were observed under light microscopy (Olympus Co., Tokyo, Japan). Pathologic lesions were scored according to the percentage of renal tubular necrosis, tubular dilation, and intratubular cell detachment present. We followed a rating schema for pathologic analysis according to previous reports: 0, normal morphology; 1, changes affecting <25% of cells; 2, changes in the range of 25–50%; 3, changes in the range of 50–75%; and 4, changes affecting >75% of the cells [29].

### 2.7. Immunohistochemistry (IHC)

After fixation and embedding, the cut kidney sections were deparaffinized and heated in citrate buffer for antigen retrieval. In order to remove endogenous peroxidase, slides were incubated with H_2_O_2_ and diluted bovine serum for 10 min and 1 h at room temperature, respectively. The following primary antibodies were added to slides for incubation at 4°C overnight: F4/80 (1 : 100, 70076, CST, MA, USA), GPX4 (1 : 100, 14432-1-AP, Proteintech, CHI, USA), or 4-HNE (1 : 100, MAB3249-SP, RD, MN, USA). These slides were subsequently treated with HRP-conjugated secondary antibody for one hour. Ultimately, the peroxidase reaction was developed with the DAB kit (ZLI-9018, Zsbio, Beijing, China), and the images were visualized under an Olympus BX51 microscope. ImageJ was used to analyze the positive areas of the IHC images. Biotinylated secondary anti-rabbit antibodies were added and incubated at room temperature for 15 min.

### 2.8. RNA Sequencing (RNA-Seq) and Analysis

To analyze the possible underlying mechanism(s) found in our study, renal tissues were collected from cisplatin-induced AKI mice that were pretreated with or without SKQ1. Total RNA isolation, cDNA library construction, and sequencing were implemented on a BGISEQ-500 RNA-Seq platform (http://www.genomics.org.cn); and initial sequence datasets were sent to the NCBI SRA database under the accession code PRJNA818125. Genes with fold change values > 2 and *P* < 0.05 were assumed to be differentially expressed. Finally, Kyoto Encyclopedia of Genes and Genomes (KEGG) and Gene Set Enrichment Analysis (GSEA) were applied to the assessment of potential signaling pathways.

### 2.9. Transmission Electron Microscopy (TEM)

Mouse renal cortex was rapidly fixed in 1.25% glutaraldehyde and postfixed in 1% osmium tetroxide, and tissue blocks were dehydrated through an ethanol gradient and embedded in Spurr's resin. Next, the ultrathin sections were generated and placed on nickel grids and sequentially stained with acidified uranyl acetate and lead citrate. The cells were ultimately photographed with an electron microscope (JEOL Ltd., Tokyo, Japan), and the percentages of damaged mitochondria were analyzed as described previously [30].

### 2.10. Analysis of Lipid Peroxidation

To evaluate total lipid peroxidation, HK2 cells were treated with cisplatin at 10 *μ*g/mL. After distinct treatments, C11-BODIPY 581/591 (D3861, Thermo Fisher, MA, USA) was applied to stain the cells according to a standardized protocol, and after incubation in the dark for 40 min, cells were redyed with Hoechst 33342 nuclear stain. Stained cells were then washed in PBS and observed using confocal microscopy (Carl Zeiss, Oberkochen, Germany). To analyze mitochondrial lipid peroxidation, cells were stained with MitoPeDPP (M466, Dojindo, Kumamoto, Japan) according to the manufacturer's protocol and mean fluorescence intensities (MFI) of oxidized MitoPeDPP were measured via flow cytometry (Beckman Coulter Life Sciences).

### 2.11. Malondialdehyde (MDA) and Glutathione (GSH) Assays

Mouse tissues or cultured cells were lysed and supernatants were collected, and we determined the levels of MDA using a specific commercial kit (S0131, Beyotime, Shanghai, China). Absorbance at 535 nm was detected by a customized microplate reader (Bio-Rad, CA, USA), and the levels of MDA were normalized per mg protein (*μ*mol/mg). The relative GSH and GSSG levels were determined using a GSH and GSSG quantification kit (S0053, Beyotime, Shanghai, China), and absorbance was recorded at 412 nm with GSH and GSSG values calculated based on a standard curve.

### 2.12. Cell Viability and Cytotoxicity Experiments

We here adopted the Cell Counting Kit-8 (KGA317, KeyGen Biotech, Nanjing, China) for viability assessments. In brief and on the basis of favorable stimulation, HK2 cells were seeded evenly on slides and 100 *μ*L of fresh medium containing 10% CCK-8 solution was added to the medium. We calculated cellular viability via their absorbance values compared to the control group. Damage to the HK2 cells was also analyzed by ascertaining the levels of lactase dehydrogenase (LDH) in cellular supernatant using an automatic biochemical analyzer (Hitachi Ltd., Tokyo, Japan).

### 2.13. TUNEL Staining

Cell death in kidney sections *in vivo* was detected using a TdT-mediated dUTP nick-end labeling (TUNEL) BrightGreen kit (A112-01/02/03, Vazyme, Nanjing, China) according to a standard procedure, and the green fluorescence of apoptotic cells was captured under a fluorescence microscope (Carl Zeiss, Oberkochen, Germany). We observed at least five random areas in each sample, and the counts of positively fluorescing cells were quantified and compared.

### 2.14. Determination of Mitochondrial Reactive Oxygen Species (mtROS) and Mitochondrial Membrane Potential (MMP)

For the evaluation of mitochondrial function, cells were seeded and treated under the designated conditions, and the levels of mitochondrially associated ROS generation were evaluated using MitoSOX-Red (M36008, Thermo Fisher, MA, USA). Cells were first incubated with 5 *μ*M MitoSOX-Red for 30 min at 37°C in the dark and then washed with PBS solution and the nuclei counterstained with Hoechst 33342. The relative fluorescence intensity was determined with a confocal laser scanning microscope or flow cytometry. Additionally, MMP levels in HK2 cells were discerned after staining with tetramethylrhodamine methyl ester (TMRM) (I34361, Thermo Fisher, MA, USA) following the manufacturer's instructions. After washing with PBS and counterstaining the nuclei, images were acquired using confocal microscopy as described above.

### 2.15. Statistical Analysis

All statistical tests were executed with unpaired Student's *t*-tests or by one- or two-way ANOVA using GraphPad Prism 6.0 software, and data are depicted as mean ± SD, unless otherwise noted. Differences between groups displaying a *P* value < 0.05 were considered to be significant.

## 3. Results

### 3.1. SKQ1 Treatment Protects against Cisplatin- (CDDP-) Induced AKI

**Figure 1:**
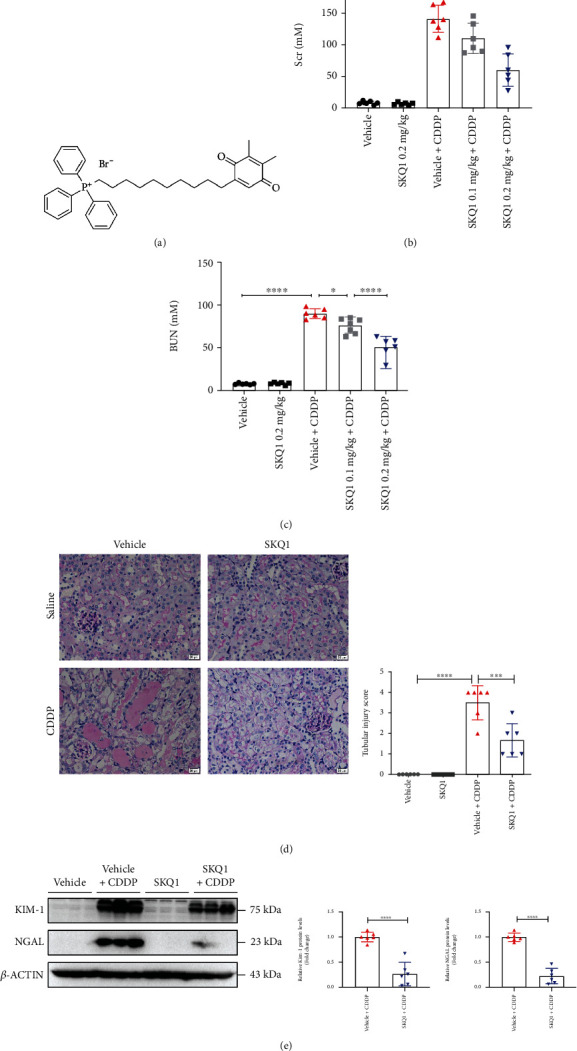
SKQ1 treatment ameliorates CDDP-induced AKI. (a) Chemical structure of SKQ1 (Visomitin). Molecular weight, 617.6; molecular formula, C36H42BrO2P. (b) SCr and (c) BUN levels of mice treated with CDDP i.p. or cotreated with various doses of SKQ1. (d) Renal microstructure upon PAS staining and renal tubular injury scores computed for all mouse groups. (e) Representative protein levels of the renal biomarkers KIM-1 and NGAL for all four groups were quantified with ImageJ (NIH, USA). The data are expressed as mean ± SD (*n* = 6). CDDP: cisplatin; ns: not significant. ^∗∗∗∗^*P* < 0.0001, ^∗∗∗^*P* < 0.001, and ^∗^*P* < 0.05 (by one-way ANOVA for (b–d), *t*-test for (e)).

SKQ1 is a mitochondria-targeting antioxidant, and its chemical structure is illustrated in Figure 1(a). To investigate the essential protective role of SKQ1 in AKI, we constructed a generic mouse model of CDDP and found that the levels of SCr and BUN were substantially elevated in the mice after CDDP administration. Intriguingly, pretreatment with 0.2 mg/kg SKQ1 markedly improved renal function by decreasing the levels of SCr and BUN (Figures 1(b) and 1(c)). We noted renal tubular detachment, dilation, and brush border damage using the PAS method and scored the degree of acute tubular injury; and the results of renal PAS staining manifested substantial histologic damage to the renal tissue as induced by CDDP—including renal tubular necrosis, dilation, and protein cast formation. These morphologic abnormalities in the kidneys induced by CDDP were, however, reversed by SKQ1 treatment (Figure 1(d)). To further substantiate the protective effects of SKQ1 against CDDP-induced nephrotoxicity, the kidney biomarkers KIM-1 and NGAL were evaluated using western blotting; and our results indicated that SKQ1 attenuated the elevated KIM-1 and NGAL protein levels observed in mice with CDDP-induced AKI (Figure 1(e)). Moreover, there were no obvious side effects of SKQ1 (0.2 mg/kg) on renal functions or other morphologic changes. Collectively, these results revealed that treatment with SKQ1 rescued CDDP-induced nephrotoxicity.

### 3.2. SKQ1 Inhibits Cisplatin-Induced Ferroptosis in the Mouse Kidney

**Figure 2:**
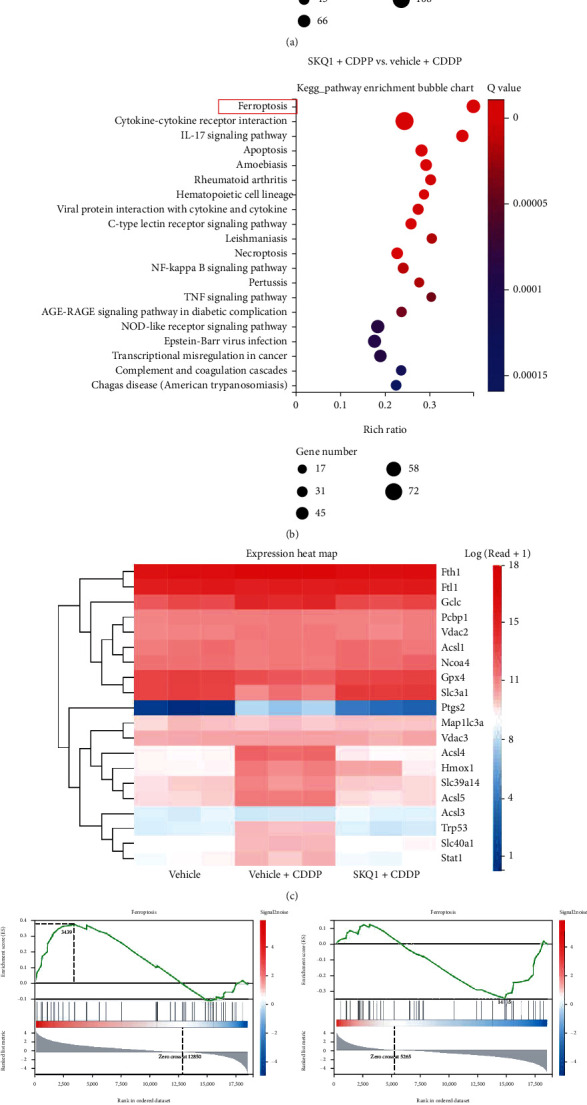
SKQ1 inhibits ferroptosis induced by cisplatin in mouse kidney. (a) Signaling pathway expression levels from samples of vehicle+CDDP and vehicle were calculated with KEGG analysis. (b) KEGG-pathway analysis of differentially expressed genes (DEGs) between the SKQ1+CDDP and vehicle+CDDP groups revealed that ferroptosis was one of the most downregulated signaling pathways (indicated by the red box). (c) A heat map of gene expression differences portrays the expressed correlations in samples among vehicle, vehicle+CDDP, and SKQ1+CDDP groups. (d) Results of the GSEA revealed that ferroptosis was upregulated in the vehicle+CDDP group compared to the vehicle group but that SKQ1 treatment markedly reduced ferroptosis compared to the vehicle+CDDP group. The expression levels of the ferroptotic regulators (e) GPX4 and (f) ACSL4 after treatment were evaluated by qPCR. Data are expressed as mean ± SD (*n* = 6 per group). CDDP: cisplatin. ^∗∗∗∗^*P* < 0.0001, ^∗∗∗^*P* < 0.001, and ^∗∗^*P* < 0.01 (by one-way ANOVA).

It was reported in several previous studies that SKQ1 functions as a mitochondria-targeting antioxidant [31, 32]. As the apparently protective mechanism exhibited by SKQ1 treatment of CDDP-induced AKI required further investigation, we performed genome-wide transcriptomic analysis and compared the differentially expressed genes (DEGs) that were upregulated after CDDP treatment of the vehicle group using KEGG-pathway enrichment.  Figure 2(a) shows that the upregulated genes were involved in cell death and principally included apoptosis, necroptosis, and ferroptosis compared to the vehicle group (indicated by the red box). Additionally, when the downregulated DEGs between the SKQ1+CDDP and vehicle+CDDP groups were also analyzed by KEGG-pathway enrichment, our results revealed that ferroptosis signaling was one of the primary downregulated pathways after treatment with SKQ1 compared to the vehicle+CDDP group (Figure 2(b)), suggesting that SKQ1 treatment inhibited CDDP-induced ferroptosis. A heat map of KEGG-pathway clustering indicated that the expression of GPX4, a negative regulator of ferroptosis, was notably reduced after CDDP administration while being markedly restored by SKQ1 treatment. Additionally, the upregulation of ACSL4 induced by CDDP was significantly inhibited by SKQ1 treatment (Figure 2(c)). GSEA results revealed that ferroptosis was greatly upregulated in the vehicle+CDDP group compared to the vehicle group and almost reversed after SKQ1 treatment (Figure 2(d)). To verify the results of RNA-Seq, relative mRNA levels for GPX4 and ACSL4 were also analyzed with qRT-PCR, and our results showed that SKQ1 treatment rescued the diminution in GPX4 mRNA induced by CDDP (Figure 2(e)). Furthermore, treatment with SKQ1 markedly reduced mRNA expression of the positive ferroptosis regulator ACSL4 (Figure 2(f)), which was then upregulated after CDDP treatment. All of these results suggest that SKQ1 inhibited ferroptosis in CDDP-induced AKI.

### 3.3. SKQ1 Treatment Attenuates CDDP-Induced Mitochondrial Dysfunction and Lipid Peroxidation-Mediated Ferroptosis *In Vivo*

**Figure 3:**
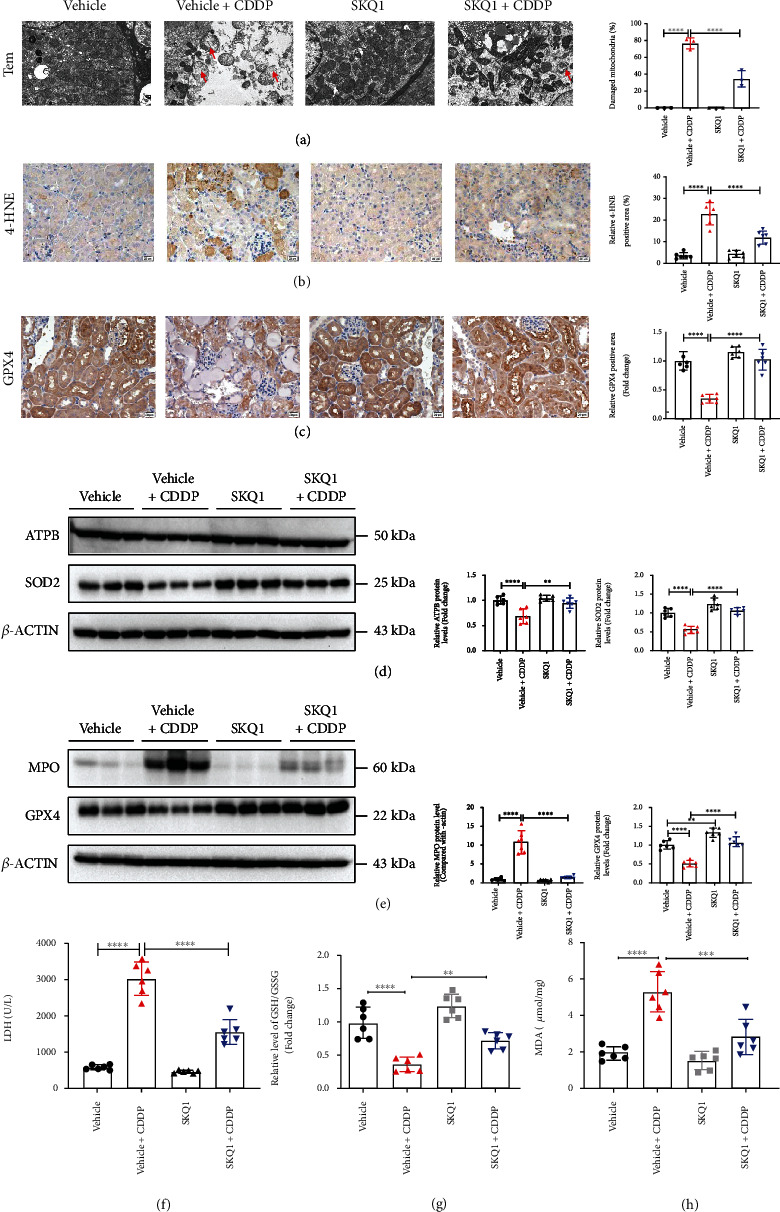
SKQ1 treatment attenuates CDDP-induced mitochondrial dysfunction and lipid peroxidation-mediated ferroptosis *in vivo*. (a) Representative TEM images of kidneys from mice treated with cisplatin with or without SKQ1 administration (the red arrow indicates injured mitochondria). IHC photomicrographs of (b) 4-HNE and (c) GPX4 in kidney tissues treated under different conditions. The protein levels for (d) ATPB, SOD2, (e) MPO, and GPX4 isolated from renal sections in all mouse groups were quantified using WB. (f) The levels of LDH in the serum of all experimental groups were determined with a commercial kit. We also analyzed the (g) relative GSH/GSSG ratio and the (h) relative levels of MDA in the renal tissue lysates. Every quantified result was expressed as the mean ± SD for each group. ^∗∗∗∗^*P* < 0.0001, ^∗∗∗^*P* < 0.001, ^∗∗^*P* < 0.01, and ^∗^*P* < 0.05 (by one-way ANOVA).

We acknowledge that ferroptosis, an iron-dependent necrosis induced by excessive lipid peroxidation, causes the unexpected loss of renal tubular cells during AKI.  Figure 3(a) depicts the percentage of CDDP-induced mitochondrial damage (another marker of ferroptosis) that was characterized by disrupted cristae, atrophy, and the rupture of the adventitia in renal tubular mitochondria as markedly alleviated by SKQ1 treatment. In this study, we determined the levels of 4-hydroxynonenal (4-HNE) and GPX4 in kidney tissues to assess iron-related lipid peroxidation and demonstrated with IHC staining that the upregulated levels of 4-HNE induced by CDDP were efficiently reduced after SKQ1 treatment (Figure 3(b)). Moreover, SKQ1 treatment restored the levels of GPX4 that were reduced in renal tubules after CDDP treatment (Figure 3(c)). SKQ1 treatment also partially restored the CDDP-induced downregulation of the mitochondrial protein components ATPB and SOD2 in renal tissues from mice (Figure 3(d)). The protein expression of myeloperoxidase (MPO) and GPX4 (markers of cellular oxidative stress and ferroptosis) was also analyzed by WB and revealed that the CDDP-induced upregulation of MPO and downregulation of GPX4 protein levels were notably mitigated by SKQ1 treatment (Figure 3(e)). In addition, the levels of lactate dehydrogenase (LDH) released after induction by CDDP were diminished after SKQ1 administration (Figure 3(f)). Upon analysis of GSH levels, we also determined that SKQ1 treatment efficiently elevated the GSH/GSSG ratio that had been significantly reduced in the kidneys of CDDP-treated mice (Figure 3(g)). The content of MDA, another marker of lipid peroxidation, also fell after SKQ1 treatment in the kidneys of mice after CDDP induction of AKI (Figure 3(h)). These results suggested that SKQ1 administration significantly ameliorated CDDP-induced ferroptosis as evidenced by a drop in lipid peroxide reactions, oxidative stress, and mitochondrial damage.

### 3.4. SKQ1 Treatment Mitigates CDDP-Induced Renal Cell Death and Inflammatory Responses

**Figure 4:**
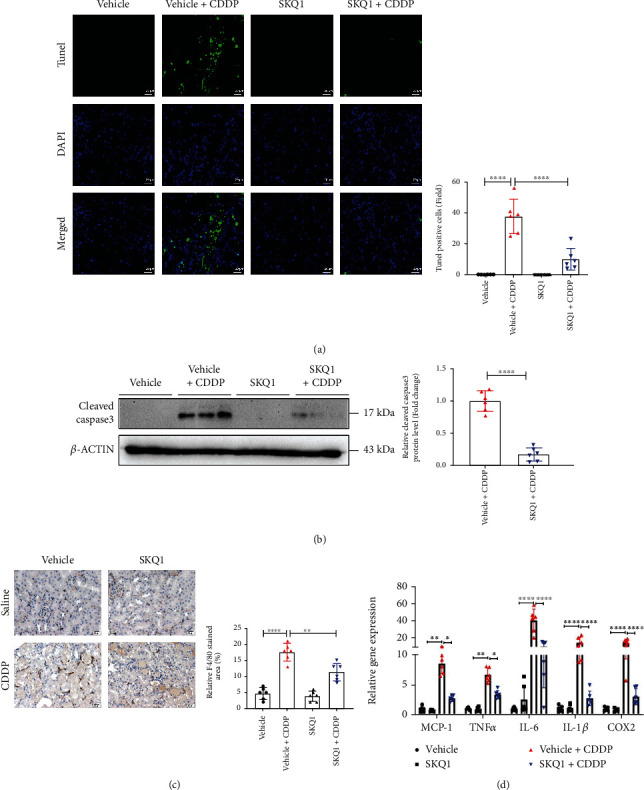
SKQ1 treatment protects against renal cell death and inflammatory responses. (a) Cell death in kidney slices was detected by TUNEL fluorescence (green, TUNEL; blue, DAPI), and numbers of TUNEL-positive cells in each group were counted. (b) Representative immunoblots of cleaved caspase-3 isolated from the same groups and relative results of densitometric analyses were evaluated. (c) We quantitatively analyzed IHC photomicrographs of F4/80 staining in renal slides treated under various conditions. (d) Mice were treated with CDDP with or without SKQ1 for the indicated times, and qRT-PCR was used to measure their MCP-1, TNF-*α*, IL-6, IL-1*β*, and COX2 mRNA levels; the results were evaluated across six different experiments. ^∗∗∗∗^*P* < 0.0001, ^∗∗^*P* < 0.01, and ^∗^*P* < 0.05 (by one-way ANOVA for (a) and (c), two-way ANOVA for (d), and *t*-test for (b)).

Although our RNA sequencing analysis showed that ferroptosis signaling was one of the main downregulatory pathways after treatment with SKQ1 compared to the vehicle+CDDP group, other pathways that included apoptosis, necroptosis, and inflammation were also downregulated after SKQ1 treatment (Figure 2(b)). To further examine the effects of SKQ1 on CDDP-induced AKI, we discerned the significant pathologic features of CDDP nephrotoxicity in cell death and inflammatory response. First, the number of TUNEL-positive cells and the expression of cleaved caspase-3 rose in renal tubules with CDDP administration but decreased after SKQ1 treatment (Figures 4(a) and 4(b)). Second, histologic analysis revealed that the number of F4/80-positive cells in CDDP-treated mice was mitigated upon SKQ1 injection (Figure 4(c)). These results thus showed that SKQ1 treatment reduced macrophage infiltration in kidneys after CDDP-induced AKI. Furthermore, the increase in proinflammatory cytokine abundance that comprised MCP-1, TNF*α*, IL-6, IL-1*β*, and COX2 in mouse kidneys after CDDP treatment was attenuated with SKQ1 (Figure 4(d)). Overall, CDDP-induced cell death and increased inflammatory response were also significantly suppressed by SKQ1 treatment.

### 3.5. SKQ1 Suppresses CDDP-Induced Mitochondrial Oxidative Stress and Ferroptosis in HK2 Cells

**Figure 5:**
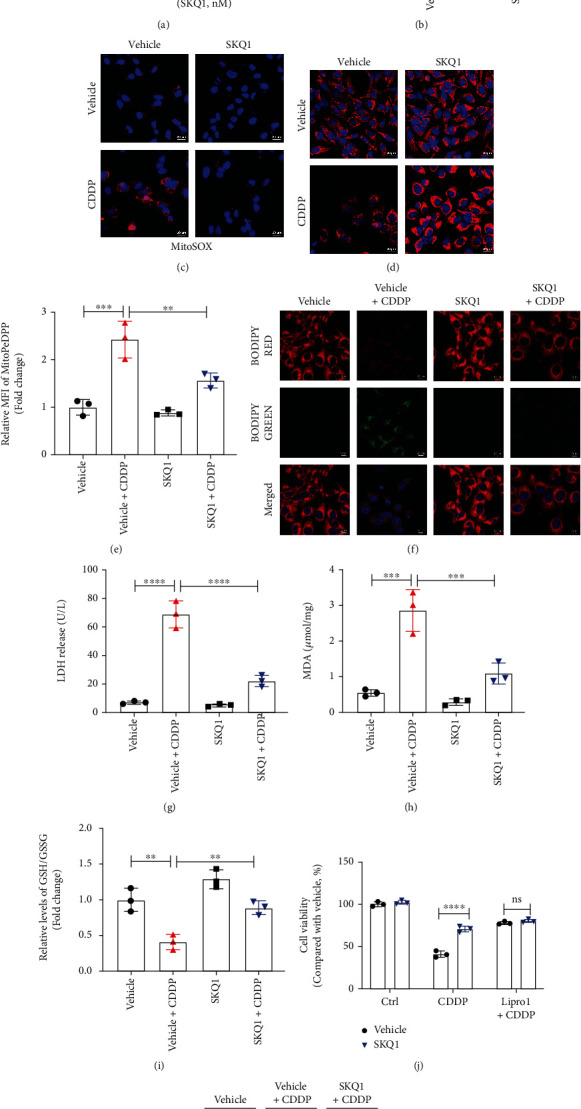
SKQ1 suppresses CDDP-induced mitochondrial oxidative stress and ferroptosis in HK2 cells. (a) Viability of HK2 cells after treatment with various concentrations of SKQ1. (b) The relative MitoSOX levels of HK2 cells treated with CDDP (10 *μ*g/mL) with or without 20 nM SKQ1 were then analyzed by flow cytometry. Representative fluorescence images of (c) MitoSOX and (d) TMRM in the four different groups of HK2 cells (red, MitoSOX or TMRM; blue, Hoechst). (e) Mitochondrial lipid peroxidation was evaluated by staining with MitoPeDPP and MFI of oxidized MitoPeDPP and analyzed using flow cytometry. (f) Representative fluorescence images of lipid peroxidation after cell staining with BODIPY dye (red, lipids; green, oxidized lipids; blue, Hoechst). (g) LDH release from HK2 cells for all groups, and (h) relative levels of MDA and the (i) relative GSH/GSSG ratio in HK2 cells treated with CDDP and cotreated with vehicle or SKQ1. (j) We applied a CCK-8 assay to ascertain the viability of HK2 cells treated with SKQ1, 10 *μ*M liproxstatin-1, or 20 mM Z-VAD-FMK, respectively, or with a combination of all three, followed by treatment with CDDP. (k) Immunoblots of GPX4 and cleaved caspase-3 in HK2 cells after treatment with CDDP and cotreatment with vehicle or SKQ1. Three independent assays were conducted. ^∗∗∗∗^*P* < 0.0001, ^∗∗∗^*P* < 0.001, ^∗∗^*P* < 0.01, and ^∗^*P* < 0.05 (by one-way or two-way ANOVA).

We employed the CCK-8 assay to analyze the *in vitro* viability of HK2 cells treated with SKQ1 at concentrations from 2.5 to 200 nM, indicating that SKQ1 concentrations not exceeding 100 nM did not influence the viability of HK2 cells relative to vehicle control (Figure 5(a)). Alterations in mtROS and MMP are common indicators of mitochondrial dysfunction, and we applied MitoSOX and TMRM red staining to evaluate mitochondrial production of ROS and MMP levels, respectively. As shown in Figures 5(b)–5(d), SKQ1 treatment attenuated mtROS generation and restored MMP downregulation induced by cisplatin treatment in HK2 cells. In addition, mitochondrial lipid peroxidation was analyzed by staining with MitoPeDPP (a mitochondria-targeting lipid peroxidation probe) and our results showed that SKQ1 treatment greatly reduced mitochondrial lipid peroxidation induced by CDDP (Figure 5(e)). Moreover, when we analyzed the total lipid peroxidation in cells by staining of BODIPY probe, we observed in the case of ferroptosis that fluorescence of BODIPY 581/591 C11 was shifted from red (reduced) to green (oxidized) under confocal fluorescence microscopy; and we also noted a red-to-green shift of BODIPY 581/591 C11 after cisplatin treatment in HK2 cells, while SKQ1 treatment reversed this shift (Figure 5(f)). Cell injury induced by CDDP was similarly assessed via the release of LDH, and the concentrations of released LDH were greatly increased after CDDP treatment and remarkably decreased after pretreatment with SKQ1 (Figure 5(g)). Analysis of other markers of ferroptosis—including the levels of MDA (Figure 5(h)) and the GSH/GSSG ratio (Figure 5(i))—indicated that abnormal changes in these markers induced by CDDP in HK2 cells were alleviated after SKQ1 treatment. We next examined the protective effect of SKQ1 treatment on the viability of HK2 cells with the CCK-8 assay, and our data showed that treatment with SKQ1, the apoptotic inhibitor Z-VAD-FMK, or liproxstatin-1, a potent ferroptosis inhibitor, notably restored the viability of HK2 cells (Figure 5(j)). However, although SKQ1 combined with liproxstatin-1 did not show an improved protective effect compared to liproxstatin-1 alone, SKQ1 combined with Z-VAD-FMK produced a superior protective effect compared with Z-VAD-FMK—suggesting that the protective effects of SKQ1 were elicited primarily by inhibiting ferroptosis in HK2 cells (Figure 5(j)). Moreover, SKQ1 treatment augmented GPX4 protein levels and reversed CDDP-induced downregulation of GPX4 in HK2 cells (Figure 5(k)). Lastly, CDDP-induced upregulation of cleaved caspase-3 protein levels was greatly inhibited by SKQ1 treatment in HK2 cells, suggesting that CDDP-induced apoptosis in HK2 cells was also inhibited by SKQ1 treatment (Figure 5(k)). Collectively, our data indicated that SKQ1 exerted its antiferroptotic action by alleviating mitochondrial oxidative stress in cisplatin-treated HK2 cells.

### 3.6. SKQ1 Treatment Circumvents FA-Induced AKI

**Figure 6:**
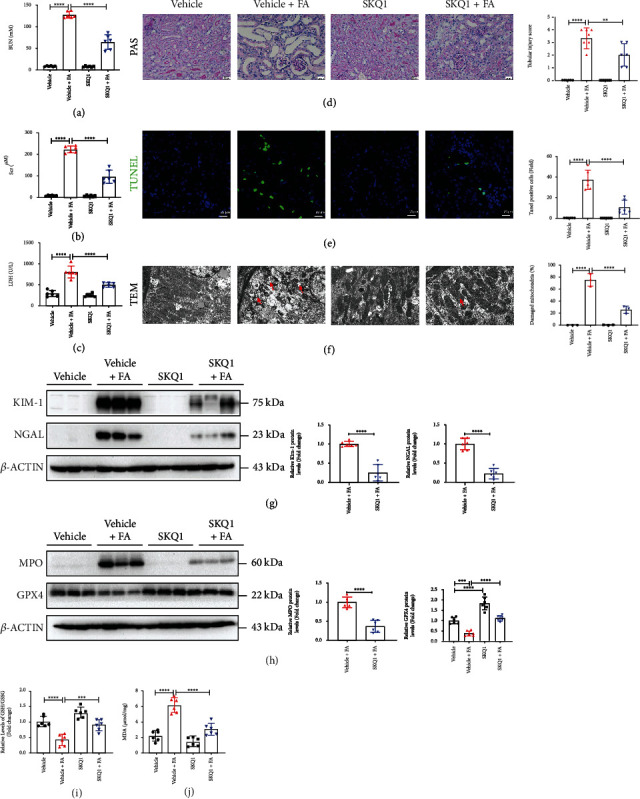
SKQ1 treatment protects against FA-induced AKI. Relative levels of (a) BUN and (b) SCr in vehicle, vehicle+FA, SKQ1, and SKQ1+FA (*n* = 6) treatment groups. (c) Serum LDH expression in all groups of the FA-induced AKI model. (d) Microstructure after PAS staining and representative renal tubular injury scores for all mouse groups were analyzed. (e) TUNEL images (green, TUNEL; blue, DAPI) and relative fluorescence intensity from each kidney were assessed. (f) Representative TEM images and quantification of damaged mitochondria (as indicated by the red arrow) in renal slides treated under various conditions. Representative immunoblots of (g) KIM-1, NGAL, (h) MPO, and GPX4 isolated from the same groups, and the relative results of densitometric analysis were evaluated; relative levels of the (i) GSH/GSSG ratio and (j) MDA for each group of FA mice with or without SKQ1 administration (*n* = 6). ^∗∗∗∗^*P* < 0.0001, ^∗∗∗^*P* < 0.001, and ^∗∗^*P* < 0.01 (by one-way ANOVA).

Considering that ferroptosis was vital to the pathogenesis of folic acid- (FA-) induced nephrotoxicity, we next explored whether SKQ1 treatment protected against FA-induced AKI. We similarly found SKQ1 treatment to be effective in restoring kidney function as evidenced by attenuated levels of the kidney injury markers serum BUN, SCr, and LDH compared with the FA-treated group (Figures 6(a)–6(c)). Histologic staining and analysis showed that SKQ1 treatment greatly mitigated morphologic abnormalities of tubular injury caused by FA-induced nephrotoxicity (Figure 6(d)) and reduced the number of TUNEL-positive cells induced by FA (Figure 6(e)); we also observed a protective effect of SKQ1 on mitochondria using TEM and calculated the percentage of damaged mitochondria. Figure 6(f) represents a lowered percentage of damaged mitochondria in the SKQ1+FA groups compared to the FA groups. SKQ1 treatment additionally substantially reduced KIM-1 and NGAL protein levels that were previously greatly induced in the FA-AKI mice (Figure 6(g)). SKQ1 treatment also upregulated the expression of GPX4, which was reduced in the kidneys of FA-treated mice (Figure 6(h)). In agreement with an increase in the protein levels for GPX4, our results showed that SKQ1 treatment effectively raised the GSH/GSSG ratio, which had previously decreased in the FA-induced AKI mouse model (Figure 6(i)), while SKQ1 significantly downregulated MPO protein levels that were upregulated in renal tissues of FA-treated mice (Figure 6(h)). Additionally, the enhanced expression of MDA in the kidneys of the FA-induced mouse model was downregulated by SKQ1 treatment (Figure 6(j)). Collectively, our data suggested that SKQ1 treatment protected against FA-induced ferroptosis and AKI.

### 3.7. SKQ1 Treatment Attenuates Renal Ischemia-Reperfusion Injury

**Figure 7:**
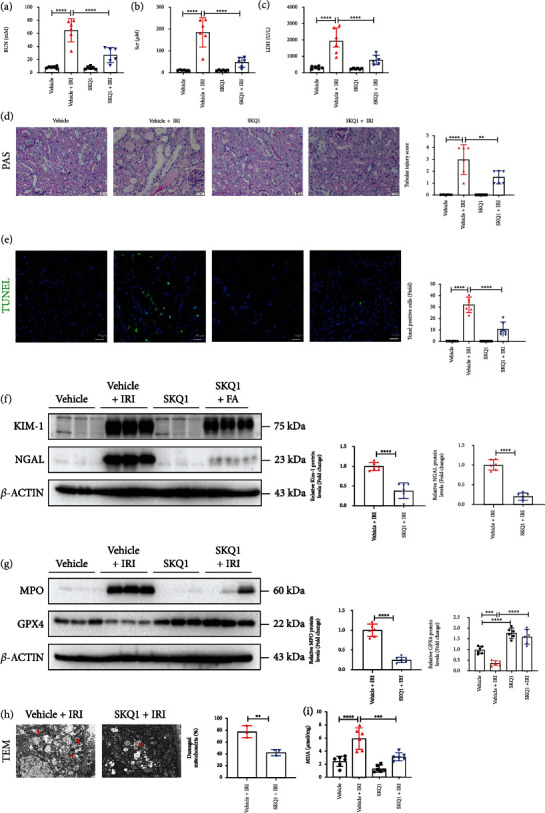
SKQ1 treatment attenuates renal ischemia-reperfusion injury. Relative levels of (a) BUN and (b) SCr in vehicle, vehicle+IRI, SKQ1, and SKQ1+IRI (*n* = 6) groups. (c) Serum LDH expression in all groups of IRI models. (d) Microstructure after PAS staining and representative renal tubular injury score for all mouse groups were analyzed. (e) TUNEL images (green, TUNEL; blue, DAPI) and relative fluorescence intensity from each kidney were determined. Representative immunoblots of (f) KIM-1, NGAL, (g) MPO, and GPX4 isolated from the same groups and relative results of densitometric analysis. (h) TEM images and quantification of damaged mitochondria (indicated by red arrow) in vehicle+IRI and SKQ1+IRI (*n* = 3) groups. (i) Relative expression of MDA in each group of IRI mice with or without SKQ1 administration (*n* = 6). ^∗∗∗∗^*P* < 0.0001, ^∗∗∗^*P* < 0.001, and ^∗∗^*P* < 0.01 (by one-way ANOVA).

To produce renal ischemia-reperfusion injury, mice underwent bilateral renal ischemia or sham surgery for 30 min with or without SKQ1 treatment, and we observed that the upregulated levels of BUN, SCr, and LDH in the serum of IRI mice were reduced by SKQ1 treatment (Figures 7(a)–7(c)). Indices of tubular injury in IRI-induced AKI—i.e., histologic tubular injury score (Figure 7(d)), TUNEL-positive cell number (Figure 7(e)), and immunoblot analysis of KIM-1 and NGAL (Figure 7(f))—were dramatically improved after SKQ1 treatment. To examine the levels of ferroptosis *in vivo*, SKQ1 treatment restored GPX4 protein levels that had been reduced after IRI, while the expression of MPO in the kidneys of the SKQ1+IRI group was considerably lower than that in the kidneys of the IRI group (Figure 7(g)). We also noted that SKQ1 improved mitochondrial morphology by TEM and reduced the percentage of damaged mitochondria in renal tubules after IRI (Figure 7(h)). The increased expression of MDA in the kidneys of mice with IRI-induced AKI was likewise decreased after SKQ1 treatment (Figure 7(i)). Collectively, these results provided evidence that SKQ1 treatment attenuated IRI-induced ferroptosis and renal injury.

## 4. Discussion

**Figure 8:**
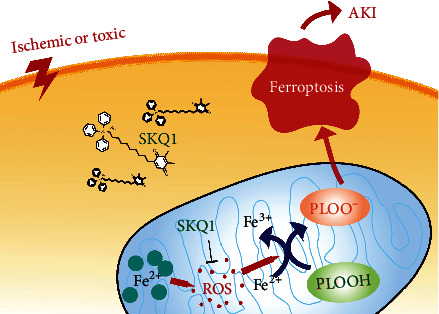
Schematic diagram of the results in the present study. Model of how SKQ1 inhibits ferroptosis and ameliorates acute kidney injury.

The pathogenesis of AKI remains incompletely understood, and there are currently no satisfactory therapies that can be applied to the treatment of AKI in the clinical setting. Although preventing tubular cell death is considered to be an effective renal protective modality for AKI [33–35] and previous studies have displayed the renoprotective function of SKQ1 in the IRI model [36], the effect of SKQ1 on toxicity-induced AKI and its precise mechanism(s) of action in protecting against AKI remain elusive. In this study, we ascertained that SKQ1 treatment not only protected against IRI-AKI but also against cisplatin- or folic acid-induced AKI. Based on RNA-Seq analysis, we provided evidence that the beneficial effect of SKQ1 was primarily elicited by inhibiting ferroptosis of tubular cells. In this regard, the present study showed that an inhibition of ferroptosis with SKQ1 treatment could be achieved via its antioxidant action and by eliminating overproduction of mitochondrial ROS, thus restoring mitochondrial morphology in renal tubular epithelial cells and protecting against AKI (Figure 8).

Inhibiting ferroptosis has been identified as a prospective therapeutic direction in renal diseases, particularly in those conditions characterized by tubular necrosis that involved toxicity- and IRI-induced AKI [37, 38]. Distinct from other types of necrosis, ferroptosis is caused by the deactivation of crucial metabolic processes that results in iron-catalyzed, lipid ROS-related cellular collapse [39]. Ferroptosis can be mediated by an exogenous or transporter-regulated pathway and an endogenous or enzyme-dependent channel [40]. GPX4 is a pivotal enzyme in the conversion of toxic lipid hydroperoxides to nontoxic lipid alcohols and is considered a major controller of ferroptosis. Deactivation of GPX4 via the suppression of ACSL4 or deletion of GPX4 leads to overwhelming lipid peroxidation and cell death [41]. We herein analyzed several markers of ferroptosis—including the GSH/GSSG ratio; the levels of MDA, MPO, 4-HNE, GPX4, and ACSL4; and the mitochondrial damage noted in several mouse AKI models—and our data revealed that the abnormal changes observed in the renal tissues of AKI mice were robustly ameliorated with SKQ1 treatment. We also found that SKQ1 treatment *in vitro* significantly reduced cisplatin-induced cell death in HK2 cells. Thus, our study extended the protective actions of SKQ1 in IRI-AKI and cisplatin- and folic acid-induced AKI.

Biologic ROS generation is derived from the mitochondria [42], and numerous studies have revealed that mitochondrion-mediated ROS production plays important roles in DNA stress, metabolic reprogramming, and lipid peroxidation in several pathologic conditions [19, 43]. Under pathologic conditions, mitochondrial ROS overproduction promotes proinflammation, apoptosis, necroptosis, and ferroptosis, ultimately contributing to tubular injury [44]. Preventing the overproduction of mitochondrial ROS may therefore be therapeutically relevant to the treatment of many diseases (including AKI) in which mitochondrial dysfunction constitutes an early event [45–48]. Additionally, a large number of studies have reported early use of antioxidants to inhibit oxidative stress response, which is beneficial to the kidney [49, 50].

SKQ1 is known as a mitochondria-targeting antioxidant capable of scavenging ROS and oxygen free radicals in a variety of diseases [51]. In the present study, while the RNA sequencing analysis showed that ferroptotic signaling was one of the chief downregulated pathways after treatment with SKQ1, we noted that the other pathways of apoptosis, necroptosis, and inflammation were also downregulated after SKQ1 treatment in mice. Several investigators previously demonstrated that mitochondria were key to mediating the crosstalk between ferroptosis and apoptosis [52, 53] and also that the inhibition of ferroptosis blocked apoptosis and necroptosis [54]. In addition, our results showed that treatment with SKQ1 combined with liproxstatin-1 did not provide greater protection compared to liproxstatin-1 alone, but that SKQ1 combined with Z-VAD-FMK facilitated improvement relative to Z-VAD-FMK alone; this suggested that the protective actions of SKQ1 were exerted via inhibition of CDDP-induced ferroptosis in HK2 cells. Our research therefore resolutely indicated that SKQ1 promoted mitochondrial homeostasis so as to mitigate AKI-associated ferroptosis. In clinical studies, SKQ1 has been proved to be safe and effective in patients with dry eye syndrome [55]. However, further experimental and clinical trials are intensely needed to confirm the nephroprotective roles of SKQ1 treatment.

## 5. Conclusions

Our collective results demonstrated a potent protective effect of SKQ1 against cisplatin-, FA-, and I/R-induced AKI. Furthermore, we demonstrated that the renoprotective effects of SKQ1 were perhaps principally mediated by inhibition of ferroptosis due to elimination of overproduction of mitochondrial ROS induced by injury *in vivo* and *in vitro*, suggesting that SKQ1 constitutes a promising candidate agent in the treatment of clinical AKI.

## Data Availability

The data used to support the findings of this study are included within the article.

## Abbreviations

AKI: Acute kidney injury
IRI: Ischemia-reperfusion injury
SKQ1: Visomitin
ROS: Reactive oxygen species
CDDP: Cisplatin
TEM: Transmission electron microscopy
MDA: Malondialdehyde
GSH: Glutathione
GSSG: Glutathione disulfide
TUNEL: Terminal deoxynucleotidyl transferase-mediated dUTP nick-end labeling
TMRM: Tetramethylrhodamine methyl ester
SCr: Serum creatinine
BUN: Blood urea nitrogen.

## Supplementary Materials

Supplementary Materials Table S1: the primer sequences of qRT-PCR.
