# Supplementary material for: Mitochondrial Targeted Antioxidant SKQ1 Ameliorates Acute Kidney Injury by Inhibiting Ferroptosis
Source: Oxid Med Cell Longev. 2022 Sep 22;2022:2223957. doi: 10.1155/2022/2223957 (PMC9526623; doi:10.1155/2022/2223957)
Supplement: Supplementary Materials — Table S1: the primer sequences of qRT-PCR. [file 2223957.f1.docx]

**Supplementary** **Table S1. Primer Sequences**

| Gene | Primer Sequence (5’-3’) |
| --- | --- |
| Mouse MCP-1 | F: GCTCTCTCTTCCTCCACCAC  R: ACAGCTTCTTTGGGACACCT |
|  |  |
| Mouse IL-1β | F: ACTGTGAAATGCCACCTTTTG  R: TGTTGATGTGCTGCTGTGAG |
|  |  |
| Mouse IL-6 | F: ACAAAGCCAGAGTCCTTCAGAGAG  R: TTGGATGGTCTTGGTCCTTAGCCA |
|  |  |
| Mouse TNF-α | F: TCCCCAAAGGGATGAGAAG  R: CACTTGGTGGTTTGCTACGA |
|  |  |
| Mouse Cox-2 | F: AGGACTCTGCTCACGAAGGA |
|  | R: TGACATGGATTGGAACAGCA |
| Mouse ACSL4 | F: CTCACCATTATATTGCTGCCTGT  R: TCTCTTTGCCATAGCGTTTTTCT |
| Mouse GPX4 | F: TGTGCATCCCGCGATGATT  R: CCCTGTACTTATCCAGGCAGA |
|  |  |
| Mouse β-actin | F: GAGACCTTCAACACCCCAGC  R: ATGTCACGCACGATTTCCC |
